# Advancing Health Equity in Digital Mental Health: Lessons From Medical Anthropology for Global Mental Health

**DOI:** 10.2196/28555

**Published:** 2021-08-16

**Authors:** Ellen Elizabeth Kozelka, Janis H Jenkins, Elizabeth Carpenter-Song

**Affiliations:** 1 Department of Anthropology University of California San Diego La Jolla, CA United States; 2 Department of Psychiatry University of California San Diego La Jolla, CA United States; 3 Department of Anthropology Dartmouth College Hanover, NH United States

**Keywords:** qualitative methods, digital health, mental health, health equity

## Abstract

Digital health engenders the opportunity to create new effective mental health care models—from substance use recovery to suicide prevention. Anthropological methodologies offer a unique opportunity for the field of global mental health to examine and incorporate contextual mental health needs through attention to the lived experience of illness; engagement with communities; and knowledge of context, structures, and systems. Attending to these diverse mental health needs and conditions as well as the limitations of digital health will allow global mental health researchers, practitioners, and patients to collaboratively create new models for care in the service of equitable, accessible recovery.

## Introduction

The past year has seen a dramatic shift in the use of digital health with the rapid adoption of telehealth within mental health settings in response to COVID-19 [1]. It has accelerated processes of patient and provider acceptance of this form of care as well as the institutionalization of digital health within the US health care system [2,3]. There is a growing evidence base regarding the potential for digital mental health [3,4]. At the same time, some scholars have raised concerns regarding the growth of digital health, noting privacy concerns and potential commodification of marginalized populations [5] along with socioeconomic and structural limitations to access [6,7]. As such, researchers and practitioners are experiencing a critical moment of opportunity to gain a deeper understanding of the promise, perils, and limitations for digital mental health. In this viewpoint, we aim to illuminate how the skills and orientations from a medical anthropological approach provide crucial insights into the development and implementation of digital health tools in the service of supporting equitable and meaningful recovery in the field of global mental health. Specifically, we argue that medical anthropology’s emphasis on illness experience; engagement with communities; and context, structures, and systems offer lessons for the field of digital mental health.

## Attention to Lived Experience of Illness

Psychological and medical anthropology have a long history of investigating cultural conceptions of mental health and illness as well as their relationship to health-seeking behaviors (eg, [8-10]). Cultural conceptions of illness are the “common sense” knowledge used to interpret experience [11]. This framework has shown that biomedical and psychiatric understandings of illness and treatment exist alongside social, spiritual, and supernatural explanations of distress as well as diverse modalities to ameliorate suffering [eg, 12-14]. Cultural conceptions of mental health and illness must be understood because they play a significant role in shaping health-seeking behaviors, treatment options, therapeutic experience, and recovery [15-17].

Attending to lived experiences and understandings of mental illness offer insight into people’s expectations and desires in relation to the various therapeutic models and goals presented in digital mental health tools. For example, an ethnographic study on community-based, residential substance use treatment in the United States-México border found spontaneous incorporation of existing digital tools to aid recovery [18]. However, the problems experienced in relation to substance use and the desired solutions could not always or easily be understood through the current biomedical framework. People and their families were seeking spiritual healing for sins or mutual aid on their recovery journey [18]. If a research team were to design novel digital mental health tools for this community, a purely biomedical framework would not fully reflect local conceptions of illness or the majority of available treatment options in Tijuana; only 8.5% of the centers self-identified their treatment as secular and clinical [19]. Ethnographic methods allow research teams to understand the ways people experience mental illness and how they may incorporate digital tools to foster meaningful mental health recovery in ways that match their desires and needs.

Qualitative and ethnographic methods facilitate engagement with first-person perspectives of mental illness and treatment. This approach goes beyond user-centered design [20,21]. Ethnography for digital mental health focuses on understanding more than just the design, acceptability, and functionality, although those are key elements. To bring in ethnographic methods refocuses attention on what it actually feels like to experience and move through the world with mental illness as well as what people’s ideas and goals are for recovery, driving innovation toward health equity. This methodology provides deeply contextualized understanding through intensive engagement with individuals and communities rather than a rapid assessment of needs and experiences that may not capture the full context of illness experience. First-person perspectives garnered through ethnographic methods thus provide opportunities to observe if and how technologies are used and made meaningful by people with mental illness, families/caregivers, and care providers.

Ethnographic methods are thus critical for the design and adaptation of digital mental health interventions. Automatic reminders from smart watches to “breathe” do little to create mindfulness habits if the barrier to care is belief (meditation will not help me) rather than memory (I keep forgetting to meditate). Better ethnographic or on-the-ground knowledge of care needs are crucial to informing the best possible avenues for aid (eg, access to smartphones, language options, stigma reduction, incorporating cultural understandings of health and illness, facilitating familial/social support systems, etc). In other words, ethnographic, qualitative methods provide a richer perspective of the fundamental human processes related to mental health, illness, and recovery in context [22], which will positively shape the design and utility of digital tools.

## Engagement With Communities

Although digital health offers a critical opportunity to decrease the global burden of mental illness, its content and form must equally reflect the conceptions of illness and priorities of people within the target population alongside evidence-based models for care. Qualitative methods are often applied to examine acceptability in the development and evaluation of digital health interventions [23]. A study examining people’s perspectives on and experiences with technology in a community mental health context found high interest in using technology as well as experience using existing smartphone features and mainstream apps to support mental health recovery [23]. For example, patients described using the alarm function as a medication reminder and daily affirmations on Instagram as helpful supports. Through close attention to first-person perspectives, scholars can better understand how underserved and marginalized populations conceptualize their illness and recovery processes to cocreate meaningful digital health services that address their real needs. As such, researchers can harness existing technology to their advantage as well as diminish redundancies by not designing products or apps with limited novel benefits to what people already have [24].

Brewer et al [25] call for community engagement to build technology that responds to the particular needs and desires of minoritized communities. Cocreating the infrastructure for its inclusion could allow vital mental health care to reach the most marginalized populations. Investment in robust digital health systems now will result in broader, long-term access to high quality health care that responds to community needs [2]. Although access and utilization have been increasing steadily even among the most marginalized [26], anthropological methods offer critical insight into the lived experience of mental illness and its intersections with technology in the service of treatment and recovery (eg, [27]).

More research is needed to understand how underserved and marginalized populations conceptualize mental illnesses and digital modalities of treatment as well as frame expectations of and motivations for seeking care. If this work is overlooked, then any scaling up of treatments may fail to result in increased utilization of services since those services will not match desired care. Frank [28] highlights that current models of addiction treatment individualize and depoliticize addiction, separating it from the context in which it occurs as well as the drivers for entering care. We argue that incorporating ethnography and other participatory methods into the design, implementation, evaluation, and adaptation of digital mental health will help address the research to practice gap [29,30].

Further, community-based participatory research can help conceptualize new models of care and recovery that address current problems of equity in mental health care. Including minoritized people with mental illness through participatory methods would allow for digital mental health to mitigate both the power differentials in health care that seem to ignore or devalue patient experiences and perspectives [31,32] as well as racial and ethnic disparities in access to health care systems [33]. These methodologies should include care providers and interdisciplinary researchers along with patients. Digital health creates opportunities for all stakeholders to participate in events they otherwise could not, such as seminars or conferences out of state aimed at shaping best care practices. However, participatory methods must go beyond incorporating people in settings where their insights have historically been marginalized. Expanding digital options to better suit the context of care will allow digital health to “bend the curve,” [2] accelerating access to high quality mental health care that suits the beliefs, needs, and recovery goals of all mental health stakeholders.

## Emphasis on Context, Structures, and Systems

Although studies suggest strong interest in using technology to support mental health among people with lived experience of mental illness, incorporating digital mental health has not been straightforward for the average person. Studies have outlined problems in uptake ranging from the overwhelming number of similar options and misalignment between application design and patient needs to the lack of an evidence base and personal data security [34,35]. Context is a key element to shaping the best, coconstructed care practices for marginalized communities (eg, care recipients, family members, Black, Latinx, indigenous, other peoples of color). This includes the cultural, structural, financial, geographic, and material differences that shape a person’s ability to encounter and engage with digital mental health. Thus, context must be the driving source of knowledge if we as global mental health researchers and practitioners hope to increase access to mental health care through digital tools.

For example, despite living on both sides of that border, from as far north as San Francisco and as far south as México City, many women in the community-based substance use treatment study previously mentioned wanted to remain in contact with their peers after they left treatment, following the 12-step orientation of the center. However, due to economic and political conditions, many of them could not travel to the center in Tijuana, México to attend weekly peer-based support meetings for former patients. As such, they began a WhatsApp group in which they hosted text message–based 12-step meetings. In this way, they were able to maintain their support networks, regardless of the type of smartphone they had or their data plans. They could participate whenever they had wireless connection available, be it at home, work, the library, or a coffee shop. Peer-to-peer care via digital platforms provides support and hope to those initially seeking out help to understand their conditions [36] as well as those whose access to their existing support community is limited by structural factors.

Throughout 2020, conversations with participants have illuminated some of the major drawbacks that center around equity in digital mental health care access. For example, many of the women in the community-based substance use treatment study did actively engage in recovery-based activities via their phones. Yet for some, their cell service was regularly turned off due to their inability to pay their bill or they were locked out of Facebook and WhatsApp accounts (primary means of communication) as abusive ex-partners hacked them. This led to women creating multiple accounts under different names, often losing touch with long-distance relatives and friends. As many of their relationships were strained due to previous or ongoing drug use, these changes or loss of contact often signaled relapse and a sharp decrease in both tangible and intangible social support. Context plays a key role in understanding the dynamic effect technologies aimed at providing aid or connection can have on people’s lives.

Similarly, another author found that strong interest in using digital health tools was tempered among people accessing community mental health programs by the limited data plans on the prepaid cell phones that were commonly owned in the population [34]. Although patients expressed interest in using new forms of technology, they were reluctant to download mental health apps that might exceed their data allotments and compromise their ability to use their phones for other needs. This underscores the critical obligation for researchers and practitioners to consider the broader context in which proposed technologies will be used and how such constraints should be foregrounded in the design and development process.

In another study led by one author focusing on adolescent mental health in southern California, the research team designed a smartphone app to help introduce adolescents to mindfulness, after discussing their needs and mental health–seeking behaviors. However, their phones would regularly be taken away by parents for a variety of reasons, from punishment to incentivizing focus on schoolwork. This meant the app, aimed at helping manage stress and anxiety, often had limited utility during periods typically associated with stress, anger, and anxiety for this particular population, mirroring findings by other teams on parental monitoring of mobile technologies by adolescents [37-39]. Cultural context thus relates to not only generalized ideas about digital health but also the dynamic social interactions that shape access to digital health tools.

If the aim of digital health is to expand care to underserved populations, then that care must take into account their differential access. Although inequitable access to mobile phones and other technology has been narrowed for those with mental illness in the United States and some other places in the world [40,41], unreliable access to the internet or continually disrupted phone service due to economic precarity still limits the feasibility of these models of care, typically for those who need it most [42]. Further, even as clinicians aim to help, differential patterns of mental health diagnoses across racial and ethnic groups change the tools people receive and the treatments they are offered, ultimately sharpening gender, class, and racial inequities in care [43,44]. As Hansen [44] aptly argues, “before we talk about how psychiatry can address the social determinants of health, we have to ask how psychiatry itself already is a social determinant of health.” Digital mental health can only address inequities in access if researchers and care providers confront how many identity groups (eg, race, ethnicity, gender, sexual orientation) are structurally marginalized within health care. Torous and Roberts [6] have called for ethical guidelines to understand shifting care needs. We similarly urge for cultural guidelines that do not ignore the very real differences in framings for mental health and necessary care. All stakeholders can then help shape novel treatments to meet their needs from the outset.

## Digital Health Aimed at Equity

Digital health has the potential to mitigate power differentials as well as disparities in mental health care systems. Qualitative and ethnographic methods have the ability to inform research and practice for novel mental health care strategies by illuminating how, in what ways, and for whom interventions work [45,46]. Understanding why people choose (or not) to use digital mental health tools will aid attempts by scholars and practitioners to make sense of the ways people evaluate care options. Further, ethnographic methods can help understand how all health care models, including novel ones such as digital mental health, assume certain values and methods for care based on cultural context [47]. In this viewpoint, we seek to point out that these assumed values may not be shared by the diverse populations that digital mental health is intended to serve. Using certain methodologies such as user-centered design [20,21] is a necessary step in mitigating this. However, our call to incorporate ethnography extends beyond the creation of an app. An app alone without community involvement in the design along with research commitment to long-term community care will not change the conditions that increase the risk for mental illness among the most marginalized. Using ethnographic methods in digital mental health projects may help sidestep the individualization of structural inequities as well as increase partnerships to improve both individual health and broader social conditions, in line with both health equity and social justice.

We believe that the conceptual and methodological approach of medical anthropology provides a strong foundation for understanding people’s dynamic conceptions of mental illness and experiences within therapeutic models; bridging gaps in understanding that can exist between care providers, patients, family members, and other stakeholders through community engagement to adapt the best possible collaborative care aimed at health equity; and broadening the focus of care and its access beyond the individual to rethink the ways social and structural barriers must be addressed when integrating novel digital health care into health systems. Medical anthropology provides a framework and methodology through which culturally humble [48,49] research and practice aimed at health equity can be conducted to incorporate digital health into the field of global mental health.
